# Anisotropic dehydration of hydrogel surfaces

**DOI:** 10.1007/s40204-017-0075-9

**Published:** 2017-10-23

**Authors:** Georgia Kaklamani, David Cheneler, Liam M. Grover, Michael J. Adams, Spiros H. Anastasiadis, James Bowen

**Affiliations:** 1 Institute of Electronic Structure and Laser, Foundation for Research and Technology Hellas, Heraklion, Crete Greece; 2 Engineering Department, Lancaster University, Bailrigg, Lancaster, LA1 4YR UK; 3 School of Chemical Engineering, The University of Birmingham, Edgbaston, B15 2TT UK; 4 Department of Chemistry, University of Crete, Heraklion, Crete Greece; 5 Department of Engineering and Innovation, The Open University, Milton Keynes, MK7 6AA UK

**Keywords:** Alginate, Dehydration, Hydrogel, Polysaccharide, Skin, Stratification

## Abstract

Efforts to develop tissue-engineered skin for regenerative medicine have explored natural, synthetic, and hybrid hydrogels. The creation of a bilayer material, with the stratification exhibited by native skin, is a complex problem. The mechanically robust, waterproof epidermis presents the stratum corneum at the tissue/air interface, which confers many of these protective properties. In this work, we explore the effect of high temperatures on alginate hydrogels, which are widely employed for tissue engineering due to their excellent mechanical properties and cellular compatibility. In particular, we investigate the rapid dehydration of the hydrogel surface which occurs following local exposure to heated surfaces with temperatures in the range 100–200 °C. We report the creation of a mechanically strengthened hydrogel surface, with improved puncture resistance and increased coefficient of friction, compared to an unheated surface. The use of a mechanical restraint during heating promoted differences in the rate of mass loss; the rate of temperature increase within the hydrogel, in the presence and absence of restraint, is simulated and discussed. It is hoped that the results will be of use in the development of processes suitable for preparing skin-like analogues; application areas could include wound healing and skin restoration.

## Introduction

Biological tissues are highly organized and stratified structures. Complex tissue architectures are composed of different cell types with specific functions and locations. The interaction between them is conducted via their extracellular matrix. The interfaces between tissues are also complex and not easily distinguishable (Dormer et al. 2010). Tissue engineering aims to develop and mimic such architectures in vitro; various methods having been developed to simulate tissue complexity and allow interaction between different cell types, proteins, implanted materials, and scaffolds (Lee et al. 2009). Methods include 3D printing, electrospinning, and cell-sheeting engineering, often requiring complicated manipulations or lengthy constructions (Grossin et al. 2009).

Skin, the largest organ of the body, exhibits a stratified and organized structure, providing a protective layer with multiple functions (Horch et al. 2005). Nerve fibres and sensory receptors permit the detection of touch, pain, and temperature (Adams et al. 2007; Adams et al. 2013). There is significant interest in developing skin analogues suitable for replacing real skin, accelerating wound healing, restoring burns, and functioning like native skin (MacNeil 2007).

Skin tissue is composed of two layers: epidermis, a waterproof barrier that excludes microbes and retains body fluids; and dermis, beneath the epidermis, which is a collagen-rich connective tissue (Hunt et al. 2009). Melanocytes impart skin colour and are found at the lower level of the epidermis, and fibroblasts are found at the dermal layer and are responsible for the strength of the skin (MacNeil 2007; Bannasch et al. 2003). Epidermis, the outer skin layer, has a surface called stratum corneum, which is a less hydrated surface layer presented at the air/skin interface. Stratum corneum is composed of dead cells formed from keratin and with thickness that varies from 10 to 15 μm for humans (Johnson et al. 1993).

Significant progress has been made in the development of tissue-engineered skin (Shevchenko et al. 2010), the skin analogue produced using cells, extracellular matrix, and combinations thereof, although the use of autografts and allografts is associated with several limitations (Priya et al. 2008; Bello et al. 2001). Various methods have been utilized to achieve skin regeneration (Yang et al. 2000; Lechler and Fuchs 2005; Fuchs and Horsley 2008), or to manufacture skin products for wound healing. Most of the skin products that have been reported use a natural, synthetic, or hybrid hydrogel as a scaffold (Priya et al. 2008; Currie et al. 2001; Bakakrishnan et al. 2005; Boucard et al. 2007; Powell and Boyce 2009), for example the use of collagen matrix encapsulated with fibroblasts and seeded with keratinocytes (Yang et al. 2000). Due to the poor mechanical properties and difficulties of handling collagen hydrogel, hybrid collagen/alginate scaffolds have also been produced (Kim et al. 2011). Other hybrid scaffolds for skin regeneration include chitosan–gelatin bilayers (Mao et al. 2003). Stratified materials have also been produced using polyelectrolyte multilayers alternated with cell-containing gel layers (Grossin et al. 2009).

Alginate has been used in tissue engineering for the regeneration of skin, bone, and cartilage due to its mechanical properties and cellular compatibility, having been used to support the growth of fibroblasts and keratinocytes (Hunt et al. 2009; Kim et al. 2011; Alsberg et al. 2001; Stevens et al. 2004). Alginate is a naturally occurring, non-toxic polysaccharide derived from brown algae (Augst et al. 2006). It is biodegradable and can be used to form hydrogels under cytocompatible conditions. Alginate hydrogels are formed through ionotropic gelation of dissolved alginate in the presence of multivalent cations such as Mg^2+^, Ca^2+^, Sr^2+^, and Ba^2+^ (Morch et al. 2006; Topuz et al. 2012), and can be gelled using the internal (Chan et al. 2002a, b) or the external gelation method (Hunt et al. 2010; Kaklamani et al. 2014). Alginate has also been used to produce hybrid hydrogels for tissue engineering applications (Choi et al. 1999), but no solution proposed yet fulfils all the requirements needed to accomplish functional stratified structures.

The aim of this work was to investigate processes by which hydrogels can be modified to provide a stratum corneum-like dehydrated surface layer. Using a high-temperature heat source directly in contact with the hydrogel, the effect of rapid dehydration by evaporative loss of water from the hydrogel surface was studied. The influence of temperature and contact duration was considered. The external gelation method was employed to produce hydrogel samples; alginate concentration was also varied and investigated. The purpose of this research is to develop processes which might be suitable for the preparation of skin-like analogues.

## Experimental

### Materials

All chemicals were sourced from Sigma Aldrich (UK) unless otherwise stated. Purities were > 99% in all cases. HPLC-grade H_2_O was employed throughout.

### Hydrogel preparation

Hydrogels (HGs) were prepared using a previously reported external gelation method (Kaklamani et al. 2014). Briefly, sodium alginate (NaAlg) solution was poured into a poly(styrene) mould (141.4 mm inside diameter, 9.0 mm inside height, Sterilin, UK) to a liquid height of 6 mm and allowed to gel in the presence of an aqueous solution of CaCl_2_ held at the upper and lower boundaries by porous microcellulose sheets. Prior to the addition of the aqueous NaAlg solution, stainless steel cylindrical spacers (21 mm diameter, 6 mm height, Longshore Systems Engineering, UK) were placed at 60 ^o^ intervals around the inner edge of the mould to support the upper sheet. The volume of aqueous NaAlg solution required was 81.75 mL. The upper sheet was held in place from above using a poly(styrene) support, filled with water to maintain close contact between the upper sheet and the NaAlg solution as it gelled, since some shrinkage was observed at the sample edges.

**Fig. 1:**
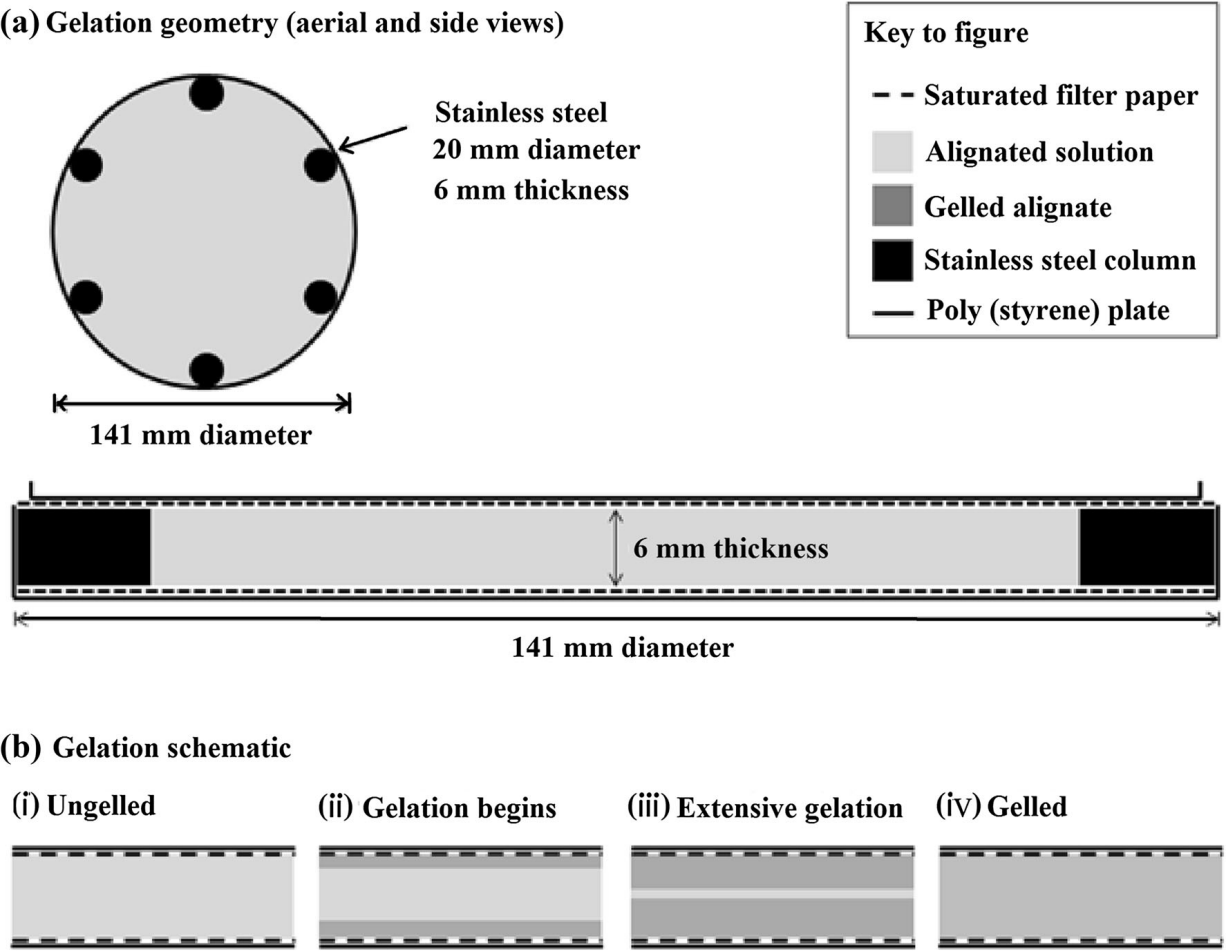
Gelation geometry (**a**) and gelation schematic (**b**) showing (i) ungelled alginate solution, (ii)–(iii) progression of the gelation through the alginate solution as the cations diffuse into the alginate solution, generating cross-links, and (iv) the final, gelled alginate. Reproduced from (Kaklamani et al. 2014)

NaAlg powder was dissolved in H_2_O under agitated conditions at a temperature of 70 °C and stirred for a minimum of 2 h using a Stuart US-152 hot plate stirrer (Appleton Woods, UK). The concentration of the NaAlg solution was 2.5% (w/v) and 5.0% (w/v). An aqueous CaCl_2_ solution of 2 M concentration was created by dissolving CaCl_2_ powder in water, whilst stirring, at room temperature; the solution was allowed to cool to room temperature of 18 °C prior to use. Microcellulose sheets (QL100, Fisherbrand, UK) were trimmed to match the shape of the poly(styrene) mould and immersed in aqueous CaCl_2_ solution for 5 min immediately prior to use. Samples were allowed to gel at 18 °C for 60 min. The gelation geometry and schematic are shown in Fig. 1, reproduced from (Kaklamani et al. 2014). The solid gels which formed are hereafter referred to by their Young’s modulus, which have also been previously reported (Kaklamani et al. 2014), and are 189 ± 25 kPa (2.5% w/v) and 433 ± 17 kPa (5.0% w/v).

### High-temperature dehydration

**Fig. 2:**
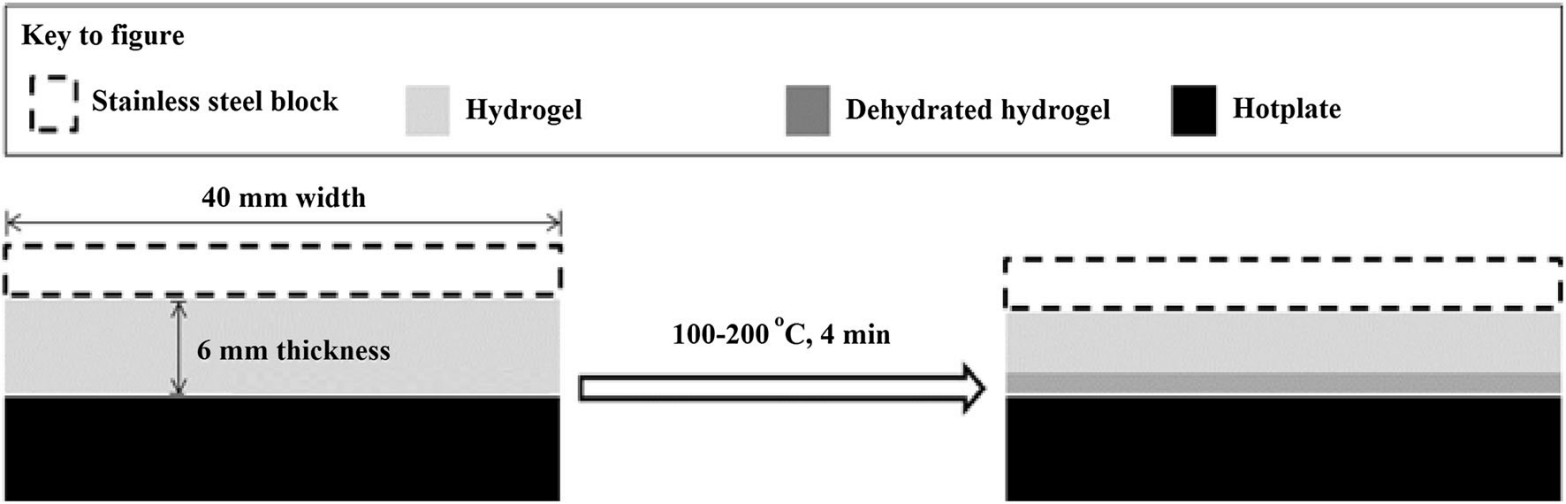
Schematic showing the dehydration process. HGs are placed in contact with a hot plate at 100–200 °C for 4 min. Specimens were either restrained, using a stainless steel block, or unrestrained

Following HG preparation, rectangular specimens (40 mm × 40 mm) were extracted using a cutting tool. HG specimens were heated for 4 min using a hot plate (Fisher Scientific, UK) at temperatures of 100, 150 and 200 °C, i.e., above the boiling point of water. Two different conditions were investigated—restrained and unrestrained. In the restrained condition, a 40 mm diameter stainless steel block (mass = 80 g) was used to ensure intimate contact between the specimen and the hot plate. In the unrestrained condition, samples were prone to migrate across the surface of the hot plate, a thin layer of water vapour lubricating the contact. A schematic is shown in Fig. 2. The face of the specimen adjacent to the hot plate is hereafter referred to as the Dehydrated Surface, while the opposite face is referred to as the Upper Surface. The mass of each specimen was measured before and after dehydration.

### Puncture testing

Puncture testing was performed at 18 °C and 40% relative humidity using a Z030 mechanical tester (Zwick/Roell, UK). A stainless steel needle (gauge 19G, bevel point, Fisher Scientific, UK) was attached to a 5 N load cell. The puncture procedure involved approaching the needle towards the HG at a velocity of 5 mm/s. The specimen was oriented with the Dehydrated Surface presented upwards, facing the needle. Load–displacement data were recorded at 100 Hz.

### Friction measurements

Tribological measurements were performed on the Dehydrated Surface using a custom-built tribometer (Longshore Systems Engineering, UK). Tests employed a rotating stage travelling at 1 mm/s, on which the HG was securely immobilized. A sphere-on-flat contact geometry was adopted, employing 12.7 mm-diameter spheres made of (i) glass and (ii) poly(propylene). Measurements were performed using an open loop control system. The normal load was 0.49 ± 0.05 N for all tests.

## Results and discussion

### Dehydration experiments

**Fig. 3:**
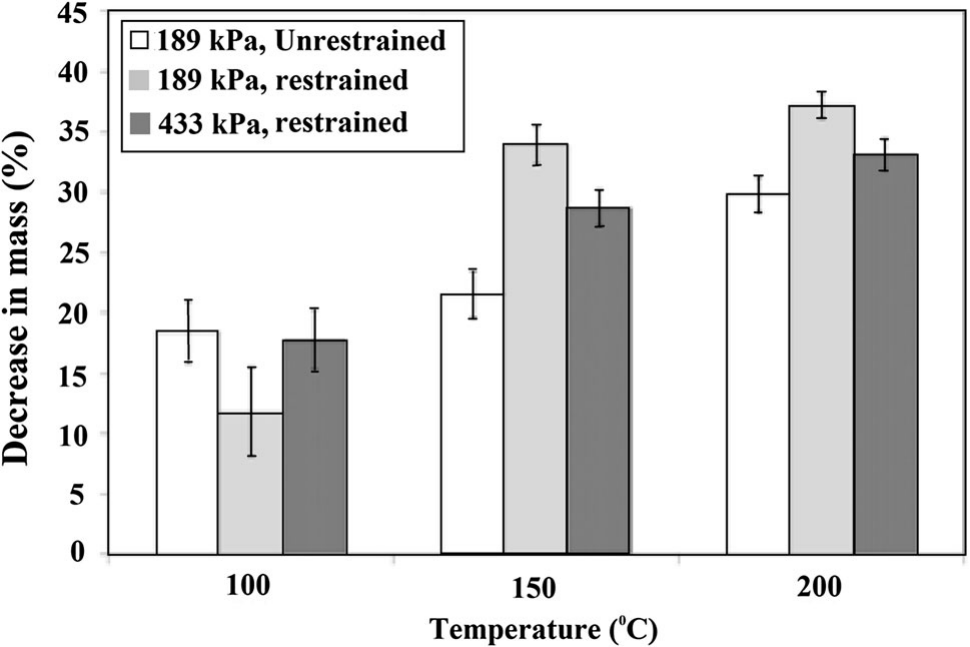
Decrease in mass for 189 kPa and 433 kPa HGs during dehydration at 100, 150, and 200 °C in the restrained and unrestrained configurations

Figure 3 shows the mass decrease for HGs dehydrated under restrained and unrestrained conditions. Increasing the dehydration temperature increases the H_2_O removed through evaporative loss, in accordance with expectations. HGs which were restrained exhibited smaller mass decreases at 100 °C than the unrestrained HGs. At 150 °C and 200 °C, however, restrained HGs exhibited larger mass decreases than the unrestrained HGs. The different rate of heat loss to the ambient atmosphere, or to the restraining disc, is thought to be the reason for the variations in mass decrease between experimental conditions. Once the restraining disc is heated sufficiently, the HG is effectively heated from both sides. This phenomenon is considered further in Sect. 3.4.

**Fig. 4:**
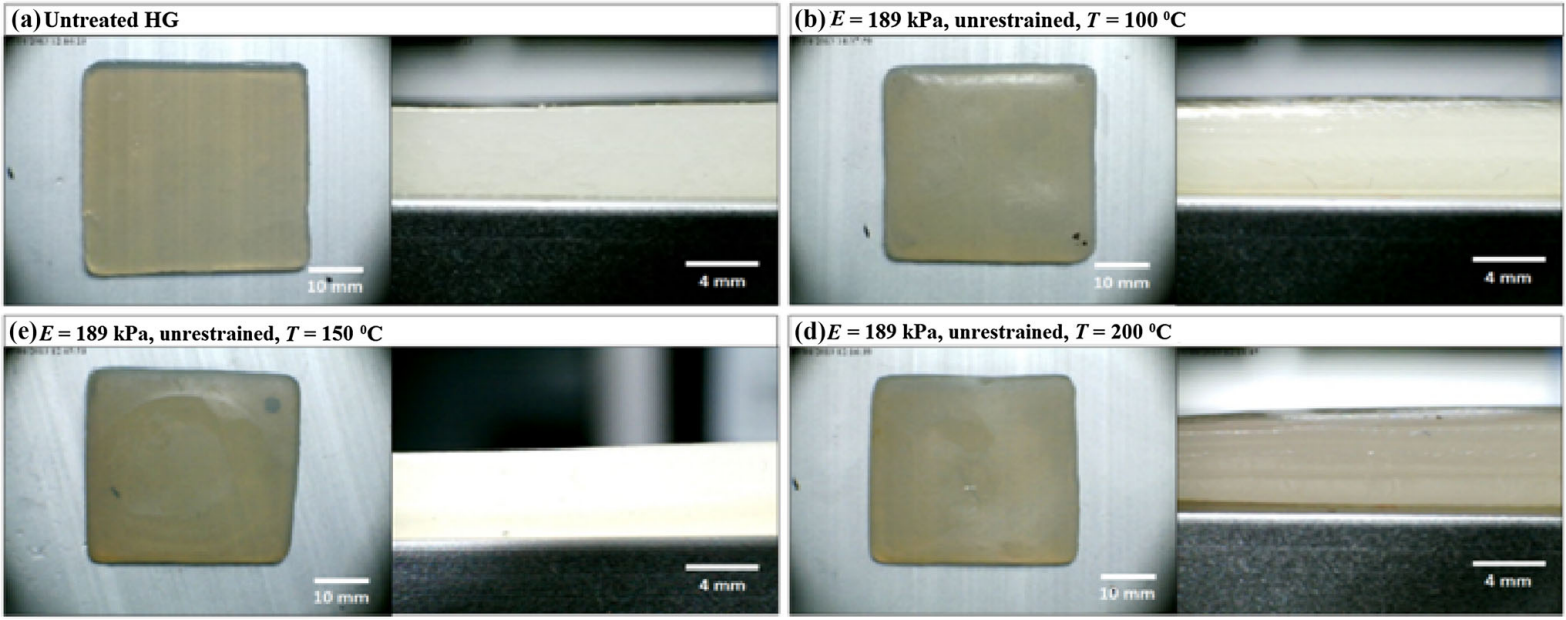
Top view and profile view for 189 kPa HGs during dehydration at 100, 150, and 200 °C in the unrestrained configuration

**Fig. 5:**
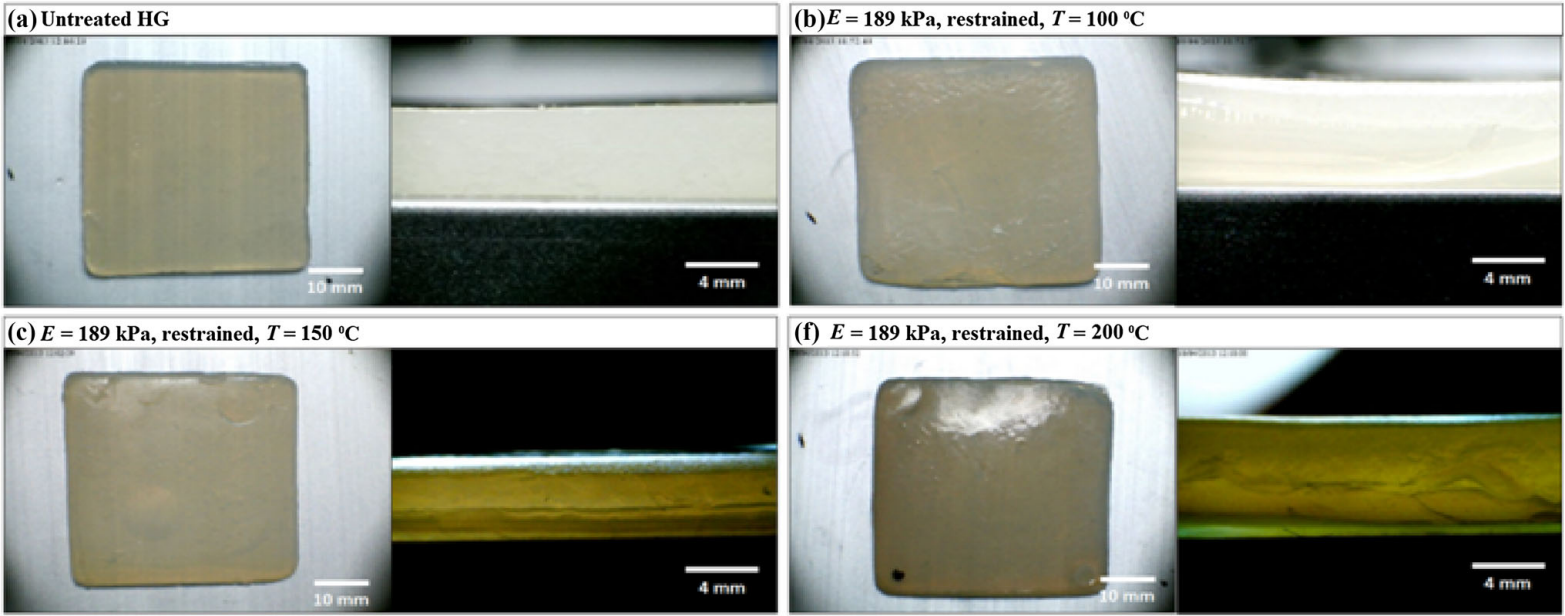
Top view and profile view for 189 kPa HGs during dehydration at 100, 150, and 200 °C in the restrained configuration

**Fig. 6:**
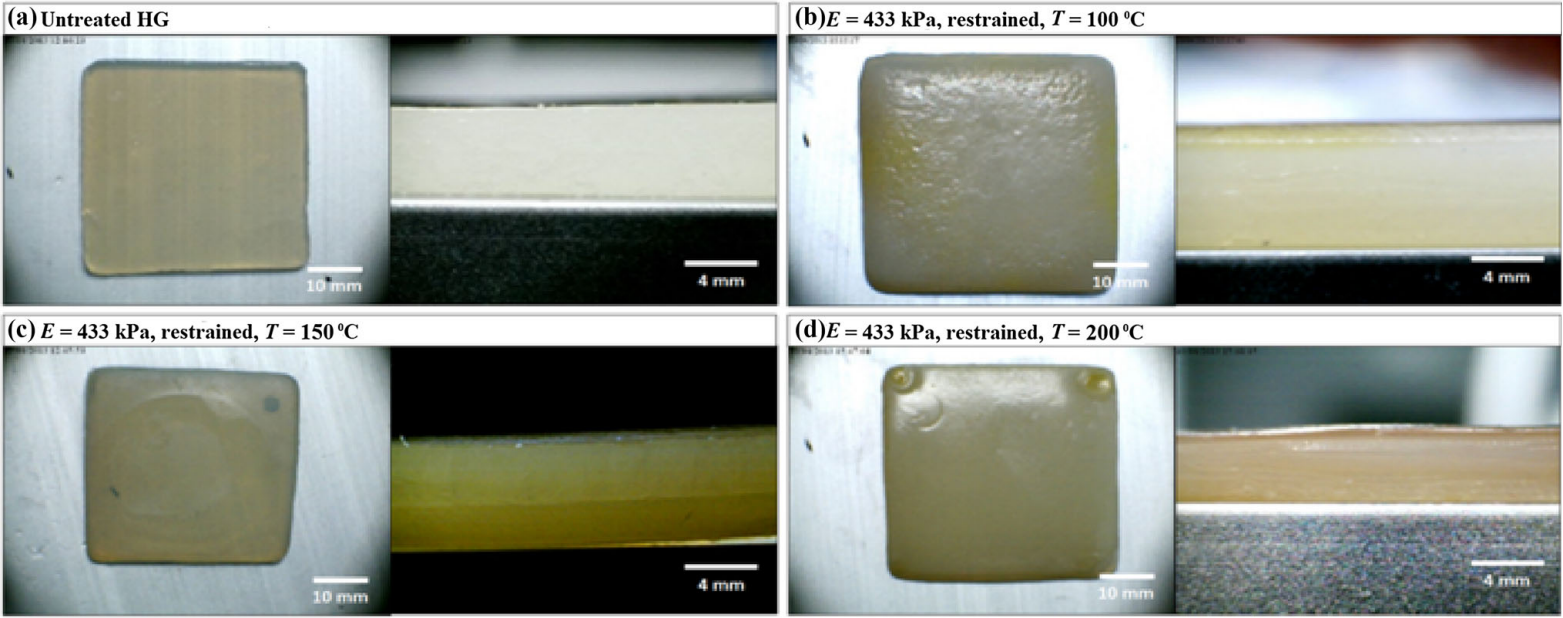
Top view and profile view for 433 kPa HGs during dehydration at 100, 150, and 200 °C in the restrained configuration

Figures 4, 5, and 6 show images of dehydrated HGs acquired using light microscopy. The profile view for the untreated HG, Fig. 4a, shows that the HG is homogeneous through its thickness. As the dehydration temperature increases, a change in the HG surface topography can be observed in the Top View images. Furthermore, anisotropic layering can be observed in the Profile View images, particularly in Fig. 5c, d.

Restraining the samples during dehydration decreased the surface area from which water could evaporate. However, the metal disc was also heated during dehydration, by heat transfer through the HG. Hence, there was unrestricted heat and vapour loss at a solid/vapour interface for unrestrained samples, but this is not true for samples which were restrained. The thickness of the dehydrated layer increases with increasing temperature, in accordance with expectations. It should be noted that exposure to temperatures higher than 200 °C tended to lead to a burnt HG surface.

### Puncture testing

**Fig. 7:**
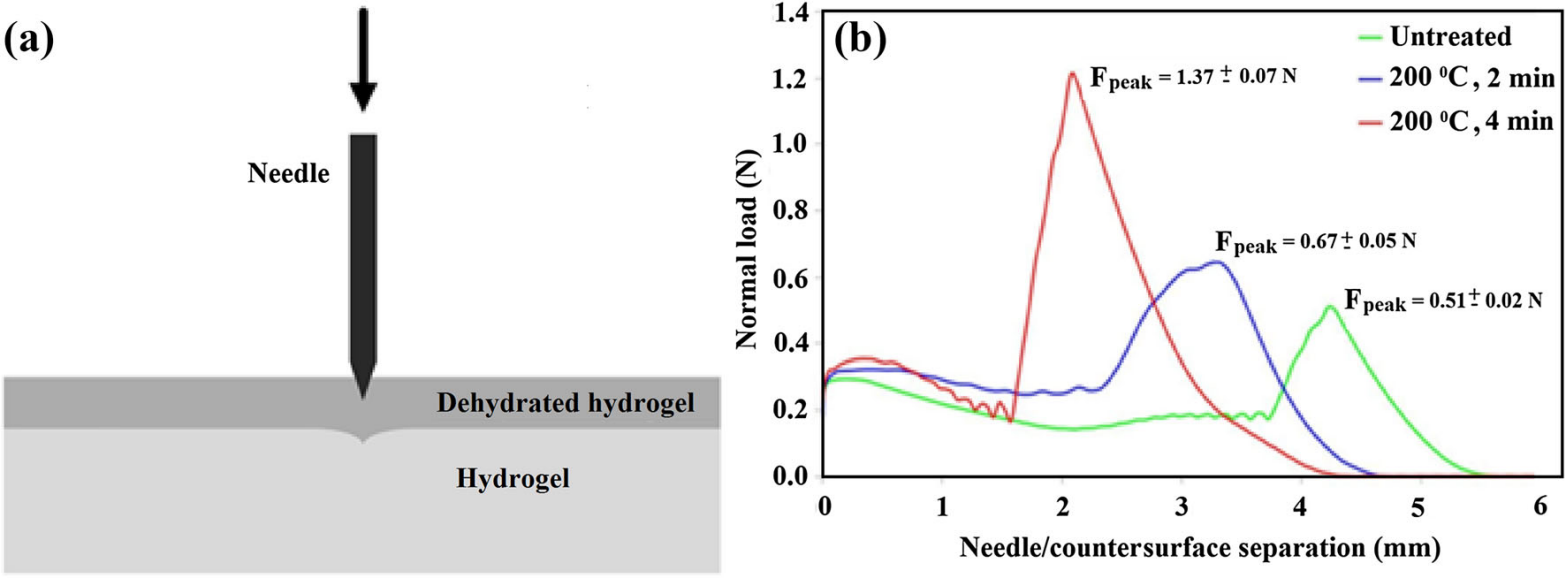
**a** Puncture schematic, showing dehydrated hydrogel as the upper surface. **b** Puncture test data for 433 kPa HGs: untreated (green); dehydrated at 200 °C for 2 min (blue); dehydrated at 200 °C for 4 min (red)

Figure 7 shows the mean peak forces for (i) the untreated HG, (ii) HG heated at 200 °C for 2 min, and (iii) HG heated at 200 °C for 4 min. Decreasing HG thickness is evidenced by the needle/counter-surface separation which decreases with increasing heating duration. Puncture tests revealed that puncture resistance increased with increasing dehydration time. The increased peak forces show that dehydration improves the mechanical properties of the HG surface. The dehydrated region of the HG exhibited a sub-surface stratification, similar to a skin-like structure. The puncture force exhibited by the dehydrated HG was in accordance with the puncture forces reported for needle insertion into skin (vanGerwen et al. 2012). The puncture resistance of HGs can be tailored by changing the cauterization time, an area for further investigation.

### Tribological testing versus glass and polypropylene

The results of tribological testing of untreated and dehydrated (200 °C, 4 min) HGs versus glass and poly(propylene) are presented in Table 1. The friction coefficient of the HG surface against both materials more than doubled due to dehydration. The glass surface is hydrophilic (*θ*_H2O_ = 0°), whereas poly(propylene is hydrophobic (*θ*_H2O_ = 112°). Hence, the HG/glass adhesion is greater than the HG/poly(propylene) adhesion, and therefore the friction coefficient is correspondingly larger for the HG/glass than for HG/poly(propylene). Further, the dehydrated surface did not visibly undergo syneresis when under contact pressure from the spherical probe, which meant that lubricating effects were negligible compared to the untreated surface. The friction coefficients of the dehydrated HG surface compare favourably with those of human skin: *μ*_glass_ = 0.26 and *μ*_PP_ = 0.35 (Adams et al. 2007).

**Table 1:** Friction coefficient of cauterized alginate against glass and poly(propylene)

Hydrogel substrate	Friction coefficient vs glass *μ*_glass_ (−)	Friction coefficient vs poly(propylene) *μ*_PP_ (−)
Untreated	0.31 ± 0.01	0.12 ± 0.01
Dehydrated, 200 °C, 4 min	0.63 ± 0.02	0.28 ± 0.01

### Effect of restraint on heating rate

A heat transfer simulation of heating the restrained and unrestrained HG samples was performed using Energy2D (v2.9.5) (Xie 2012). The material properties used are shown in Table 2. The initial hot plate temperatures of 100, 150, and 200 °C were specified; changes in sample thickness due to evaporative losses were not incorporated.

**Table 2:** Parameters used for heat transfer simulations

Material	Density *ρ* (kg m^−3^)	Heat capacity *C*_p_ (kJ kg^−1^ K^−1^)	Thermal conductivity *k* (W m^−1^ K^−1^)
Air	1.204	1.012	0.025
Aluminium hot plate^a^	2700	0.921	205
Hydrogel	1070	4.184	0.591
Steel disc	8000	0.502	50

^a^Constant temperature

**Fig. 8:**
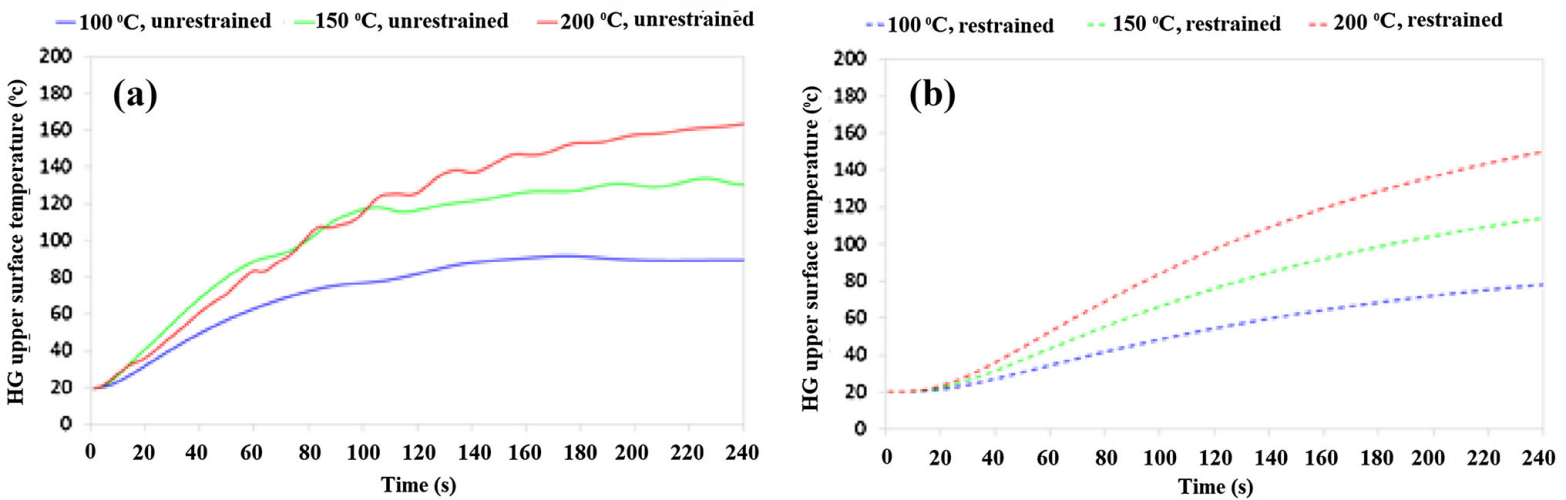
Temperature of the HG sample upper surface when heated from below using a hot plate, **a** unrestrained, and **b** restrained

Figure 8 shows the effect of incorporating the restraining disc on the temperature of the upper surface of the 433 kPa HG. The presence of the restraining disc decreases the rate of temperature increase within the HG. The disc also increases in temperature, and is typically 15–20 °C lower than the HG. In the restrained condition, energy from the hot plate accumulates in the aluminium disc and the HG. In the unrestrained condition, energy is lost more easily, because the HG is a good thermal conductor. This disattenuates the energy loss at the upper surface, by preventing heat transfer from the HG to the ambient atmosphere. The disc also means that the HG is heated from above and below when restrained.

Considering the *E* = 189 kPa HGs in Fig. 3, at *T* = 100 °C the restrained HG loses less mass than the unrestrained HG. This is because the temperature of the restrained HG increases more slowly than the unrestrained HG. The HG temperature does not reach 100 °C, the boiling point of water, for either the restrained or unrestrained condition. At *T* = 150 and 200 °C, the restrained HGs exhibit greater mass loss than the unrestrained HGs; why might this be? Here, the HG temperature rises to an excess of 100 °C for both the restrained and unrestrained conditions. It is suggested that energy retained within the restrained HG/aluminium disc increases the rate of water evaporation, which otherwise would have caused an increase in temperature. In comparison, this energy is conducted through the unrestrained HG and is lost at the HG/air interface. The difference in behaviour occurs when the hot plate temperature is increased above the boiling point of water.

## Conclusions

We report the effect of local exposure to high temperatures on the surface properties of alginate hydrogels. Rapid dehydration of the surface using temperatures in the range 100–200 °C leads to the creation of a mechanically strengthened hydrogel surface layer, exhibiting improved puncture resistance and increased coefficient of friction compared to the unheated surface. Direct contact with a surface at 200 °C for 4 min produced the most mechanically robust layer. Mechanical constraint of the hydrogel sample was important during processing, preventing curvature of the sample during cauterization, particularly at the edges.

Further investigations will study a greater variety of HG compositions, as well as explore in more detail the importance of dehydration temperature, duration, the thermal conductivity of the HG, and the nature of the restraint material. A modified apparatus for dehydration within a humidified atmosphere, for reducing evaporative loss, is currently being designed. The possible benefit of performing the heating whilst the HG is immersed in an aqueous solution, perhaps a physiologically relevant buffer, is also under consideration. Finally, an improved understanding of the material structure remaining following the dehydration process would be useful.
